# Rapid induction of complete molecular remission by sequential therapy with LDAC and sorafenib in FLT3-ITD-positive patients unfit for intensive treatment: two cases and review of the literature

**DOI:** 10.1186/1756-8722-6-39

**Published:** 2013-06-11

**Authors:** Denise Wolleschak, Enrico Schalk, Christian Krogel, Tina M Schnoeder, Helga Luehr, Kathleen Jentsch-Ullrich, Thomas Fischer, Florian H Heidel

**Affiliations:** 1 Department of Hematology and Oncology, Center of Internal Medicine, Otto-von-Guericke University Medical Center, Leipziger Str. 44, Magdeburg D-39120, Germany; 2 Private Practice for Hematology and Medical Oncology, Hasselbachplatz 2, Magdeburg D-39104, Germany

**Keywords:** AML, Sorafenib, Small molecule, LDAC, Targeted therapy

## Abstract

Treatment of acute myeloid leukemia remains a therapeutic challenge. Even in younger patients with a low rate of co-morbidities less than 50% of patients can be cured. For older patients or patients with significant co-morbidities, the situation appears even worse. In patients not eligible for intensive treatment approaches - e.g. due to underlying medical conditions - therapeutic approaches remain almost exclusively palliative. However, even with less intense treatment approaches, temporary remission can be achieved and this contributes to prolonged survival and improved quality of life of the respective patient. Targeted therapies have been widely used as palliative treatment in- and outside clinical trials as single agents. Combination with low-dose cytarabine (LDAC) potentially improves remission rates and can be safely administered in an outpatient setting.

Previous studies showed that additive hematologic toxicity of combinatory therapeutic approaches may arise from simultaneous treatment (e.g. chemotherapy plus targeted therapies). However, sequential therapies have already proven their feasibility in clinical trials. Here, we report two cases of rapid induction of complete molecular remission by sequential therapy with LDAC and sorafenib in patients unfit for intensive chemotherapy without significant long-term toxicity.

## Background

Acute myeloid leukemia (AML) is an aggressive malignant disease characterized by abnormal proliferation of immature hematopoietic cells. AML is the most frequent form of acute leukemia in adults. Despite advances in chemotherapeutic treatment within the last decade, only 30-45% of patients below the age of 60 can be cured. The majority of patients, above the age of 60, have a dismal prognosis with only 10-15% long-term survival [1-4]. While cytogenetic changes are established markers of prognosis and response [5,6], several molecular markers have been defined and analyzed for their prognostic influence [7,8]. Length-mutations (or ‘internal tandem duplications’, ITD) of the FLT3 tyrosine kinase occur in approximately one third of adult patients with AML [7-9]. Clinically, the occurrence of *FLT3*-ITD mutations in AML is associated with a higher probability of relapse and shortened disease-free- and overall-survival and therefore is considered as an unfavorable prognostic factor. *FLT3*-ITD mutations show a high degree of heterogeneity with respect to their length, the number of mutated clones, the allelic ratio of the duplicated segments and the insertion sites. These variables may have a dramatic impact on clinical outcome [10-14]. This view is supported by gene expression profiling demonstrating that *FLT3*-ITD cases are a heterogeneous entity [15].

Using myelosuppressive chemotherapy regimens, complete hematologic remission (CR) can be achieved in 60-80% of patients below the age of 60. However, the majority of patients with AML are above the age of 60, and most of these patients do not qualify for or do not benefit from myelosuppressive chemotherapy. Using myelosuppressive chemotherapy, only 38-62% of elderly patients reach CR and only 5-15% show long-term overall survival compared to approximately 40% of younger patients. The prognosis is even worse for patients who are not eligible for myelosuppressive chemotherapy due to underlying medical conditions. For those who are not eligible, less aggressive treatment approaches are clearly warranted. Using low-dose cytarabine (LDAC) as monotherapy, a complete remission (CR) rate of 17% and a partial remission (PR) rate of 19% with a median survival of 15 months could be shown in a meta-analysis [16]. As this can be administered on an outpatient basis and ‘reaching a remission’ as well as ‘outpatient treatment’ are known factors to significantly improve quality of life in patients suffering from leukemia, LDAC can be considered a feasible alternative. It has been demonstrated convincingly, that outcome of LDAC treated patients is superior to hydroxyurea treatment, however this benefit seems to be restricted to cytogenetic subgroups [17]. The advantage in overall survival corresponds to the achievement of a significant remission and achievement of a CR also impacts quality of life in a positive manner. Several studies have been conducted or are under way to determine the efficacy of targeted agents in combination not only with intensive [18-20] but also low dose chemotherapy [21,22]. Sorafenib is one example of a multikinase inhibitor targeting FLT3-receptor as well as BRAF, KIT and PDGFR that has been investigated as monotherapy and in various combination schedules. One important lesson learned from these trials is, that toxicity may arise from concomitant treatment with chemotherapy and targeted therapies. Combination of the FLT3 kinase inhibitor sorafenib with myelosuppressive [23,24] or low-dose chemotherapy [22] with overlapping dosing schedules led to increased toxicity and required reduction of therapeutic medication. In contrast, sequential therapy of chemotherapy with kinase inhibitors has already proven its feasibility including a modest and acceptable toxicity rate [23].

Recent studies have substantially supported the concept of FLT3-ITD as a valid therapeutic target in human AML, and suggested that FLT3-ITD is capable of conferring a state of ‘oncogene addiction’, whereby cellular survival pathways associated with normal or precancerous cells can become hijacked, leading to a state of reliance upon key signaling molecules that can be exploited therapeutically [24]. Therefore, targeting mutated FLT3-kinase in addition to standard chemotherapy offered the opportunity to inhibit signaling pathways crucially required for maintenance of AML. A sequential therapy schedule was chosen to avoid severe hematologic toxicity as outlined above.

Here, we report on two cases of FLT3-ITD positive AML in patients unfit for intensive chemotherapy being treated with sequential therapy consisting of LDAC followed by oral administration of sorafenib resulting in rapid induction of complete molecular remission with no significant toxicity arising.

## Case report 1

**Figure 1:**
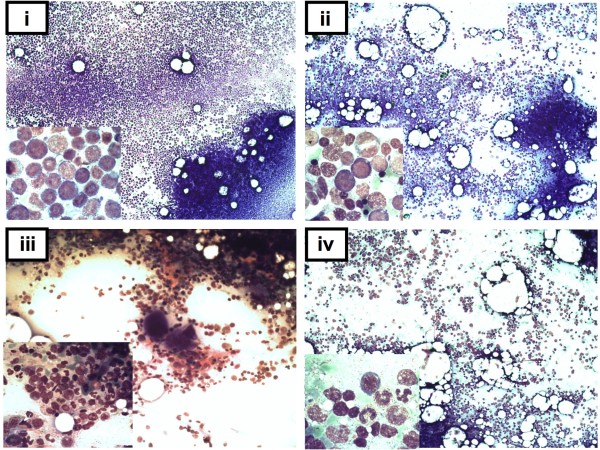
**Cytomorphology of bone marrow (BM) aspirates at diagnosis and at remission controls obtained after the first cycle of sequential therapy. Case 1** (left column): (i) Aspirate at primary diagnosis showing 80% BM infiltration with myeloid blasts. The malignant cells show large nuclei, narrow cytoplasm and fine azurophilic granula. (iii) Complete remission (CR1) achieved after one cycle of low-dose cytarabine (LDAC)/sorafenib. Differentiating granulopoiesis and reappearance of megakaryopoiesis as observed after reconstitution of peripheral blood counts. **Case 2** (right column): (ii) Predominant expansion of the erythroid lineage with pronounced dysplasia. The non-erythroid cells show 67% of myeloid blasts consistent with the diagnosis of erythroleukemia (FAB M6). (iv) Reduction of cellularity and reconstitution of granulopoiesis following the first cycle of sequential LDAC/sorafenib therapy. Achievement of complete hematologic remission with concomitant reconstitution of peripheral blood counts.

**Figure 2:**
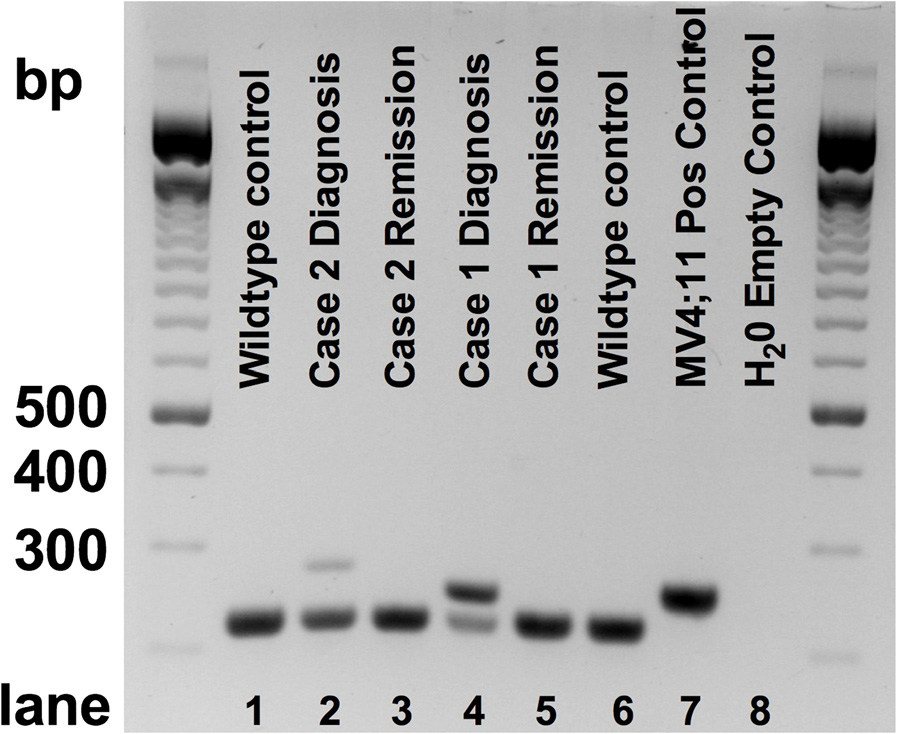
**PCR analysis at primary diagnosis and remission.** Standard diagnostic PCR was performed on DNA derived from bone-marrow aspirates at primary diagnosis and remission. The PCR assay used allows a sensitivity above 1:100 (as tested using MV4;11 cells as a positive control in comparison to healthy donor PBMC). *FLT3*-ITD mutations were detectable in samples of both patients at diagnosis (lane 2 - patient 2 and lane 4 - patient 1). Complete molecular remission could be confirmed in both remission samples (lane 3 - patient 2 and lane 5 - patient 1). MV4-11 cells served as positive control (lane 7) and healthy donors or water as negative controls (lane 6 and 8).

A 74-year old woman was referred from a county hospital to our department in January 2013 with leukocytosis, anemia and thrombocytopenia. She was in no apparent distress, however, presented on admission with reduced overall physical fitness and kachexia (consistent with an ECOG-score of 2). Laboratory findings presented as follows: Platelets (PLT) 92 Gpt/l hemoglobin (HGB) 10.6 g/dl and white blood count (WBC) 42.30 Gpt/l, with 5% neutrophils, 7% lymphocytes, 1% eosinophils and 81% myeloid blasts. Bone marrow aspiration confirmed the replacement of normal hematopoiesis by blasts of myeloid morphology, FAB M2 (Figure 1, upper left panel). Karyotype analysis revealed a normal female karyogram (46,XX) while molecular diagnostics was positive for an FLT3-length mutation (FLT3-ITD) (Figure 2, lane 4). Her physical examination was normal except a harsh 4/6 systolic murmur spreading to the carotid arteries and over the precordium. Echocardiography revealed a dilated left atrium and mild hypertrophy of the left ventricle while demonstrating good ventricular function (LVEF = 65%). The aortic valve appeared highly stenotic (planimetric 0.9 cm^2^) and the mitral valve presented with a modest stenosis of 1.8 cm^2^). Taken together, our patient was not eligible for intensive, myelosuppressive chemotherapy, as the necessary volume of infusion therapy may have resulted in pulmonary edema and congestive heart failure.

**Figure 3:**
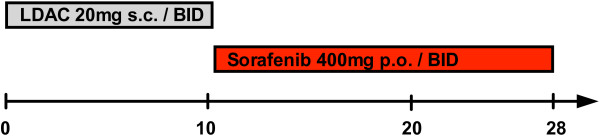
**Treatment schedule.** Sequential treatment with low-dose cytarabine (LDAC) 20 mg s.c. BID was conducted for 10 consecutive days followed by sorafenib 400 mg p.o. BID days 11–28.

Therefore, we initiated palliative treatment using LDAC at a dose of 20 mg BID for 10 days, followed by sorafenib 400 mg BID days 11–28 (Figure 3). The patient developed severe (grade 3–4) but transient pancytopenia of less than 7 days and received six red blood cell transfusions and three transfusions of platelets. By day 28 white blood count and platelets appeared to be normalized with regular differentiation. Bone marrow aspiration performed on day 28 (following the first cycle) showed regeneration of the hematopoiesis with slight dysplasia but complete disappearance of myeloid blasts (Figure 1, lower left panel). PCR amplified FLT3-wildtype alleles with the mutated alleles no longer detectable (Figure 2, lane 5). The patient remained in complete molecular remission with dependency on red blood cell transfusions during the LDAC-administration period.

## Case report 2

A collaborating rehabilitation center referred a 58-year old woman having suffered from a massive stroke of the left hemisphere with consecutive right hemiplegia and aphasia. In the past medical history, the husband reported heavy smoking, arterial hypertension, hyperlipoproteinemia, diabetes and overweight of his wife for several years resulting in myocardial infarction 10 years ago and consecutive congestive heart failure. The massive stroke became evident just weeks before admission. During rehabilitation blood counts were taken and revealed profound anemia. The physiatrist referred the patient to our department for red blood cell transfusion and diagnostic procedures.

On admission we saw an adipose woman with pronounced hemiplegia, aphasia (consistent with an ECOG score of 3). She appeared pancytopenic with a WBC of 1.61 Gpt/l, HGB of 9.5 g/dl and PLT count of 71 Gpt/l.

Bone marrow aspiration revealed pronounced hyperplasia of erythropoiesis with severe signs of dysplasia (Figure 1, right upper panel). Among the non-erythropoietic cells we found 67% myeloid blasts, thus confirming diagnosis of acute erythroleukemia (AML FABM6). The aspirate showed a normal female karyotype (46,XX) and a PCR positive for an *FLT3*-ITD mutation (Figure 2, lane 2).

In consent with the patient and her husband we initiated palliative treatment using LDAC at a dose of 20 mg BID for 10 days, followed by sorafenib 400 mg BID days 11–28 (Figure 3). During the first cycle of LDAC the patient remained leukopenic and developed neutropenic fever, which resolved after empiric antibiotic treatment with meropenem. Peripheral blood counts improved stepwise during the second cycle of LDAC/sorafenib and the peripheral blood counts normalized 6 weeks after treatment initiation. Bone marrow aspiration confirmed CR by cytomorphology (Figure 1, lower right panel) and complete molecular remission (Figure 2, lane 3) by PCR. The patient is currently under ongoing therapy in an outpatient setting with a relapse free survival of more than 290 days.

## Discussion & review of the literature

Currently, sorafenib is not licensed for treatment of acute myeloid leukemia (AML) but rather for treatment of hepatocellular carcinoma or renal cell carcinoma. As a consequence, sorafenib is not routinely prescribed on an outpatient basis for AML. Despite these difficulties, several reports have been published using sorafenib mostly as a palliative treatment approach for relapsed and refractory AML with a certain variability in response rates and mostly hematologic toxicity.

Sorafenib has been investigated repeatedly as a single agent for myeloid neoplasia. Within an early phase I dose escalation trial 50 patients with advanced myelodysplastic syndrome and/or refractory acute leukemia (AML, n = 48) were treated with sorafenib monotherapy [25]. All patients received a starting dose of 200 mg BID in 2 treatment arms: schedule (A) 5 days per week, every week for a 21 day cycle and schedule (B) for 14 days every 21 days. The maximal tolerable dose (MTD) – due to hematologic toxicity - was found to be at 400 mg sorafenib BID. The most common side effects were fatigue (58%), nausea/vomiting (44%) diarrhea (36%) and dyspnea (30%). In terms of clinical responses, CR or CR with incomplete recovery (CRi) of platelets could be achieved in 10% of patients (n = 5). Moreover, 17 patients (34%) revealed reduction of bone marrow- or peripheral blood-blast count.

Man and colleagues recruited 13 patients with relapsed or refractory FLT3-ITD positive AML to an open-label, single-arm study [26]. Patients were treated with 200 or 400 mg sorafenib (BID) until either disease progression or eligibility for hematopoietic stem cell transplantation (HSCT). In this study, a high percentage of patients (6/13; 46%) responded with CRi. In the context of HSCT, 65 patients with relapsed, chemotherapy-refractory FLT3-ITD positive AML have been compiled for retrospective analysis [27]. Patients were categorized in two groups with one group (n = 36) receiving conventional chemotherapy and the second group (29 patients) receiving sorafenib as a single agent for relapsed AML after allogeneic HSCT. In 42 patients sorafenib was given at a standard dose of 400 mg BID. Frequently reported side effects included severe pancytopenia (grade 3–4) in 40/65 patients (61.5%). Efficacy was significantly lower compared to other reports cited with 7.7% CR, 20% CRi and 44.6% of hematologic improvement/blast response. Ten patients with a complete remission achieved also a complete molecular remission (CMR). Overall response to sorafenib was comparable in a survey analysis of 29 relapsed and refractory AML patients (with a median age of 64.5 years) treated outside of clinical trials at 30 clinical centers in Germany [28]. Here, 6/23 patients (21%) responded with CR (n = 2; 8.7%) or CRi (n = 4; 17.4%). The majority of these patients had intermediate risk cytogenetics (93%) and showed positivity for *FLT3*-length mutations (81%). Less than half of the patients were NPM1-mutated (43%). In most of the reported patients (82%) sorafenib was given as a single agent, while 18% received sorafenib in combination with LDAC. Again, most prevalent toxicities included cytopenia (in almost every patient) followed by nausea and rash. By the time of the survey, 47% of patients were alive at a median observation of 131 days. In all other patients the median overall survival was 86 days after initiation of sorafenib treatment. Overall survival appears comparable or slightly below the results of published data on palliative treatment with either hydroxyurea or LDAC [17] in patients considered unfit for intensive chemotherapy at primary diagnosis.

Combination of LDAC with different ‘small molecules’ has been assessed in some clinical trials [21,22]. The combination of LDAC with sorafenib has been recently investigated in a phase I/II trial for elderly patients with AML or high-risk MDS [22]. Here, within the phase I sorafenib was started at 200 mg BID days 2–28 followed by stepwise increase of the dose level. LDAC was given at 10 mg s.c. BID days 1–10. 13 patients were treated in phase I. Within the expansion phase II, sorafenib was given at a final dose of 600 mg days 2–28 and LDAC 10 mg s.c. BID days 1–10 to another 8 patients. Out of 15 evaluable patients two patients achieved a PR (13%) and CR (13%), respectively, and two further patients (13%) showed a stable disease. Given the combination of two effective drugs, the response rate seemed significantly lower than the ones published in previous reports, however, only 14% of patients revealed *FLT3*-ITD mutations. The most frequent non-hematologic toxicities were erythema multiforme, febrile neutropenia, hypertension, tremor and fatigue of minor severity. However, severe hematologic toxicity (grade 3–4) occurred in the vast majority of patients with 95% thrombocytopenia, 76% neutropenia and 62% anemia. This massive occurrence of hematologic toxicity led to delay or reduction of therapy and may be the consequence of increased sorafenib dosing (up to 600 mg per day) contemporaneously administered with LDAC. Taken together, response rates using sorafenib as a single agent or in combination with low-dose chemotherapy have been reported in the range of 13-46% CR or CRi, even when applied in relapsed or refractory disease.

Previous studies have investigated the relevance of molecular monitoring of FLT3-ITD mutations. Thiede and colleagues investigated a total of 979 FLT3-ITD mutated AML patients treated with chemotherapy. Here, FLT3-ITD positive patients with high mutant/wildtype allelic ratio had a dismal prognosis in regard to overall survival (OS) and disease free survival (DFS). Moreover, these patients had a significant increased probability of relapse [12]. A second study investigated in 11 cases with AML FLT3 mutations in order to assess for minimal residual disease (MRD). MRD was measured by single step real-time PCR. Five of eleven patients developed positive MRD and all five patient MRD-positive patients experienced relapse [29]. Upon sorafenib treatment, two recent reports have assessed for residual disease on a molecular level [27,30]. Taken together, detection of minimal residual disease has been assessed previously, however, not systematically upon sorafenib treatment. Patients with high mutant/wt allelic ratio treated with conventional chemotherapy seem to experience inferior overall survival (OS) and disease free survival (DFS). Positive MRD was associated with a significantly higher risk of relapse. In our hands, combined treatment with LDAC and sorafenib led to rapid induction of CMR in both patients reported. Given the available data on minimal residual disease after treatment with chemotherapy and targeted therapies, one can assume that depth of response would correlate with improved overall survival and therefore would improve quality of life. However, to the best of our knowledge, none of the previous publications investigating sorafenib for FLT3-ITD mutated AML correlated the depth of response (CMR versus CHR or PR) with overall survival and especially quality of life. We believe that rapid induction of a CMR could eventually contribute to prolonged survival and increased quality of life. From our perspective these questions need to be addressed prospectively in future clinical trials dealing with treatment of elderly, frail or clinically challenged patients.

The two patients reported by us, immediately achieved complete molecular remission after one cycle of sequential therapy, consistent with excellent response rates of FLT3-ITD positive cohorts published before. However, in the literature responses are not exclusively restricted to *FLT3*-mutated patients. In our hands this treatment approach appeared to be safe, feasible and could be administered to patients unfit for intensive chemotherapy in an outpatient setting. Both patients reported a clear benefit in quality of life and in both cases clinical responses were durable over several months.

Overall, we conclude that sequential therapy with LDAC followed by sorafenib appears to be an effective treatment for patients unfit for intensive chemotherapy. Sequential therapy was feasible even in an outpatient setting and did not show substantial hematologic toxicity as anticipated by simultaneous treatment approaches in recent clinical trials. However, prolonged treatment over several cycles may deserve dose reduction or shortage of treatment days to avoid hematologic toxicity, which is the major challenge of this treatment approach. A larger sample size of patients will have to be included in a prospective clinical trial to evaluate this treatment strategy in detail.

## Consent

Written informed consent was obtained from both patients for publication of this case report and any accompanying images. A copy of written consent is available for review.

## Competing interests

The authors declare that they have no competing interests.

## Authors’ contributions

All authors read and approved the final manuscript. ES and TF collected and provided data on the inpatient treatment of the cases presented. CK and KJU collected and provided data on the outpatient treatment of both patients presented. HL and TMS performed PCR analysis on the patient samples and documentation of bone marrow aspirates. DW analyzed data, compiled diagnostic data and contributed to the writing of the manuscript. FHH analyzed data and wrote the manuscript.
